# Random interval schedule of reinforcement influences punishment resistance for cocaine in rats

**DOI:** 10.1016/j.nlm.2024.107961

**Published:** 2024-07-16

**Authors:** Bradley O. Jones, Haley F. Spencer, Adelis M. Cruz, Morgan S. Paladino, Sophia N. Handel, Rachel J. Smith

**Affiliations:** a Institute for Neuroscience, Texas A&M University, TAMU 3474, College Station, TX 77843, USA; b Department of Psychological and Brain Sciences, Texas A&M University, TAMU 4235, College Station, TX 77843, USA

**Keywords:** Addiction, Compulsive, Footshock, Self-administration, Habit, Random ratio

## Abstract

In an animal model of compulsive drug use, a subset of rats continues to self-administer cocaine despite footshock consequences and is considered punishment resistant. We recently found that punishment resistance is associated with habits that persist under conditions that typically encourage a transition to goal-directed control. Given that random ratio (RR) and random interval (RI) schedules of reinforcement influence whether responding is goal-directed or habitual, we investigated the influence of these schedules on punishment resistance for cocaine or food. Male and female Sprague Dawley rats were trained to self-administer either intravenous cocaine or food pellets on a seeking-taking chained schedule of reinforcement, with the seeking lever requiring completion of either an RR20 or RI60 schedule. Rats were then given four days of punishment testing with footshock administered at the completion of seeking on a random one-third of trials. For cocaine-trained rats, the RI60 schedule led to greater punishment resistance (i.e., more trials completed) than the RR20 schedule in males and females. For food-trained rats, the RI60 schedule led to greater punishment resistance (i.e., higher reward rates) than the RR20 schedule in female rats, although male rats showed punishment resistance on both RR20 and RI60 schedules. For both cocaine and food, we found that seeking responses were suppressed to a greater degree than reward rate with the RI60 schedule, whereas response rate and reward rate were equally suppressed with the RR20 schedule. This dissociation between punishment effects on reward rate and response rate with the RI60 schedule can be explained by the nonlinear relation between these variables on RI schedules, but it does not account for the enhanced resistance to punishment. Overall, the results show greater punishment resistance with the RI60 schedule as compared to the RR20 schedule, indicating that schedules of reinforcement are an influencing factor on resistance to negative consequences.

## 1. Introduction

Drug addiction is characterized by compulsive drug use despite negative consequences. In an animal model of compulsive drug use, a subset of rats continues to seek cocaine despite footshock consequences and is considered punishment resistant (Belin, Mar, Dalley, Robbins, & Everitt, 2008; Deroche-Gamonet, Belin, & Piazza, 2004; Pelloux, Everitt, & Dickinson, 2007; Vanderschuren & Everitt, 2004). Punishment resistance for cocaine has frequently been investigated using random interval (RI) or variable interval (VI) schedules of reinforcement (Chen et al., 2013; Jonkman, Pelloux, & Everitt, 2012a, 2012b; Limpens, Schut, Voorn, & Vanderschuren, 2014; Limpens, Damsteegt, Broekhoven, & Voorn, 2015; Pelloux et al., 2007; Pelloux, Dilleen, Economidou, Theobald, & Everitt, 2012; Pelloux, Murray, & Everitt, 2015; Vander-schuren & Everitt, 2004; Zhou et al., 2019). Punishment resistance has also been investigated using a fixed ratio 5 (FR5) schedule of reinforcement (Belin, Berson, Balado, Piazza, & Deroche-Gamonet, 2011; Belin et al., 2008; Belin, Balado, Piazza, & Deroche-Gamonet, 2009; Cannella et al., 2013; Deroche-Gamonet et al., 2004; Kasanetz et al., 2010, 2013). However, it is unclear how ratio and interval schedules of reinforcement compare in terms of their influence on punishment resistance for cocaine. A recent study of food self-administration in mice showed increased punishment resistance with an RI60 schedule as compared to an RR20 schedule (Seiler et al., 2022). Further, it is well established that RR and RI schedules of reinforcement bias food responding to be goal-directed or habitual, respectively, and we recently showed a similar bias with cocaine responding (Dickinson, 1985; Dickinson, Nicholas, & Adams, 1983; Gremel & Costa, 2013; Hilario, Holloway, Jin, & Costa, 2012; Jones, Cruz, Kim, Spencer, & Smith, 2022; Killcross & Coutureau, 2003; Wiltgen et al., 2012; Yin & Knowlton, 2006; Yin, Knowlton, & Balleine, 2004, 2005, 2006; Yin, Ostlund, Knowlton, & Balleine, 2005). Therefore, we sought to investigate the influence of RR and RI schedules on punishment resistance for cocaine.

Habitual behavior has long been implicated in compulsive drug seeking (Belin, Belin-Rauscent, Murray, & Everitt, 2013; Brown, Dayas, James, & Smith, 2022; Everitt and Robbins, 2005, 2016; Everitt, 2014; Ostlund & Balleine, 2008; Smith & Laiks, 2018). Habitual behavior is elicited by conditioned stimuli and insensitive to outcome devaluation, whereas goal-directed behavior is performed in direct pursuit of the outcome and sensitive to outcome devaluation (Balleine & Dickinson, 1998; Handel & Smith, 2024; Yin & Knowlton, 2006). We recently showed that punishment resistance for cocaine is associated with habitual responding, i.e., insensitivity to outcome devaluation (Jones et al., 2024). Although punishment resistance was not predicted by habitual cocaine seeking, it was associated with continued use of habits, whereas punishment sensitivity was associated with increased goal-directed responding (Jones et al., 2024). Given that habits are involved in punishment resistance and that RI schedules bias habitual responding, we hypothesized that the RI60 schedule of reinforcement would lead to greater punishment resistance as compared to the RR20 schedule. In our previous study, we did not observe a significant difference between RR20 and RI60 schedules in terms of punishment resistance for cocaine when comparing the final day of punishment (Jones et al., 2024); therefore, in the current study we evaluated schedules across multiple punishment days and in larger groups of rats.

We used a seeking-taking chained schedule of reinforcement that has been used extensively to study punishment resistance (Chen et al., 2013; Datta, Martini, Fan, & Sun, 2018; Datta, Martini, & Sun, 2018; Giuliano et al., 2018; Giuliano, Belin, & Everitt, 2019; Giuliano, Puaud, Cardinal, Belin, & Everitt, 2021; Jonkman et al., 2012b; Limpens et al., 2014, 2015; Olmstead et al., 2000, 2001; Pelloux et al., 2007, 2015; Vander-schuren & Everitt, 2004; Zhou et al., 2019). Rats were assigned to either an RR20 or RI60 schedule for the seeking lever. Some of the rats in the current analyses were also included in our previously published work (Jones et al., 2024). In the previous study, we only analyzed rats that met the inclusion criteria for outcome devaluation so that we could investigate the role of habits in punishment resistance. Here, we analyzed data from a larger group of rats trained on RR20 or RI60 schedules (some of which did not meet inclusion criteria for outcome devaluation) so that we could assess the influence of schedules on punishment resistance. We found that male and female rats trained to self-administer intravenous (IV) cocaine showed greater punishment resistance with the RI60 schedule as compared to the RR20 schedule when evaluating trials completed. Female rats trained to self-administer food also showed greater punishment resistance with the RI60 schedule, although male rats showed punishment resistance with both RI60 and RR20 schedules. Interestingly, we found that punishment caused greater reductions in seeking responses as compared to trials completed for the RI60 schedule, indicating a dissociation in punishment effects on response rate and reward rate for RI schedules. Overall, these data indicate greater punishment resistance with the RI60 schedule.

## 2. Materials and methods

### 2.1. Animals

Male and female Sprague Dawley rats (initial weight 225–250 g; Charles River, Raleigh, NC, USA) were single-housed in a temperature- and humidity-controlled facility at Texas A&M University (accredited by AAALAC). Rats were housed under a reversed 12-h light/dark cycle (lights off at 6 a.m.), with food and water access ad libitum, except when noted below. All experiments were approved by the IACUC at Texas A&M and conducted according to specifications of the NIH as outlined in the Guide for the Care and Use of Laboratory Animals.

Some of the data included in the current work are from rats that were used in previously published work (Jones et al., 2024). Cocaine self-administration data includes 72 male and 48 female rats. Of those rats, 15 males and 22 females were included in the previous study, 10 males and 12 females were run after the previous work, and 47 males and 14 females were not included in the previous study because they did not receive outcome devaluation testing or did not meet the criteria for outcome devaluation inclusion (at least one of following: did not receive appropriate cocaine dose for devaluation, did not make ≥10 presses on nondevaluation session pre- or post-punishment, could not undergo post-punishment devaluation because did not recover to baseline self-administration after punishment). Food self-administration data includes 33 male and 22 female rats. All these rats were included in the previous work.

### 2.2. Surgery

Rats that were used for cocaine self-administration studies were implanted with chronic indwelling IV jugular catheters, as previously described (Smith, See, & Aston-Jones, 2009), while under isoflurane anesthesia (induction 5 %, maintenance 1–3 %) and after treatment with a nonsteroidal anti-inflammatory analgesic (ketoprofen, 2 mg/kg, s.c.). Starting three days after surgery, catheters were flushed once daily with cefazolin (0.1 ml, 100 mg/ml) and heparin (0.05 ml, 500 U/ml). Self-administration sessions began at least five days after surgery.

### 2.3. Cocaine self-administration

As described previously (Jones et al., 2024), rats were trained to self-administer IV cocaine (0.5 mg/kg per infusion) on a seeking-taking chained schedule of reinforcement, in which completion of a random ratio (RR20) or random interval (RI60) schedule on the seeking lever gave access to the taking lever during daily 2-h sessions. Operant conditioning chambers were controlled via MED-PC IV (Med-Associates, St. Albans, VT). Cocaine HCl was obtained as a gift through the NIDA Drug Supply Program and diluted in sterile 0.9 % saline.

Self-administration training began with fixed ratio 1 (FR1) reinforcement on the taking lever (criterion of 5 sessions ≥20 infusions), during which rats were mildly food-restricted (85–90 % of weight) until they met the criterion of ≥2 consecutive sessions ≥20 infusions. Rats were then moved to a chained seeking-taking schedule with FR1 requirements on both levers (criterion of 2 days ≥15 infusions). A stimulus light was on above the seeking lever when it was available. Completion of seeking led to retraction of the seeking lever and extension of the taking lever; a press on the taking lever delivered cocaine and caused retraction of the taking lever and the start of the next trial. A 4-min time out was added between trials, during which the seeking lever was extended but with no stimulus light on and no consequence to pressing (criterion of 2 days ≥15 infusions). The RR or RI schedules were then introduced for the seeking lever, and the taking lever was available for only 60 sec or until an infusion was earned, whichever occurred first. For the RI schedule, the first press initiated the start of the random interval and then the first press made following the random interval completed the schedule. Training began at RR3 or RI10 (criterion of 2 days ≥15 infusions), progressed to RR10 or RI30 (criterion of 2 days ≥15 infusions), and then to the final schedule of RR20 or RI60 (criterion of ≥13 days ≥15 infusions). The majority of rats also experienced outcome devaluation testing during this final stage of training prior to punishment, as presented in our prior work (Jones et al., 2024).

### 2.4. Food self-administration

As described previously (Jones et al., 2024), rats were trained to self-administer food pellets (45-mg plain purified pellets, Bio-Serv, Flemington, NJ) on a seeking-taking chained schedule of reinforcement (RR20 or RI60) similar to the training for cocaine self-administration except that the criterion was 3 days = 30 pellets for FR1 taking, 2 days ≥20 pellets for FR1 seeking-taking, and ≥12 days ≥20 pellets for the final schedule of RR20 or RI60; criteria for other training stages was similar. The time out between trials was only 1 min and the seeking lever was retracted. Additionally, sessions were limited to 1 h or 30 rewards, whichever occurred first. Rats were mildly food-restricted for the entire experiment but still gained weight. Feeding occurred in the home cage each day >1 h after the self-administration session ended and was individualized per rat; each rat was fed the maximum amount that resulted in no remaining food before the next day’s session (~50 g for males, ~20 g for females). The majority of rats also experienced outcome devaluation testing during this final stage of training prior to punishment, as presented in our prior work (Jones et al., 2024).

### 2.5. Footshock punishment

Rats received four days of punishment, during which footshock (0.4 mA, 0.3 sec) was administered on 1/3 of trials randomly, after completion of the seeking link. After the first trial (which was never given footshock), the MED-PC program selected one of every three trials for footshock by randomly selecting one value per trial from an array of three values without replacement, such that one value could not be reselected until the other two values were selected (e.g., 1, 2, 3, 2, 3, 1, 1, 3, 2…, with a value of 1 indicating footshock on that trial). The taking lever and rewards were still available on footshock trials.

### 2.6. Estrous cycle evaluation

Estrous cycle was evaluated via vaginal smears and cytology. Females were swabbed daily on several days before punishment and through punishment testing. A cotton swab wet with filtered deionized water was used for vaginal swabbing and then smeared onto a glass slide. Estrous cycle was categorized as proestrus, estrus, metestrus, or diestrus phase according to the proportions of cells, as described by Ajayi and Akhigbe (2020).

### 2.7. Data analyses

Animals were removed from analyses if they did not meet the minimum self-administration criteria after two weeks at a given stage of training. Statistical analysis was conducted using GraphPad Prism software (version 10). Unless noted otherwise, data were analyzed using t-tests or 2-way or 3-way ANOVAs (with repeated measures when appropriate) as detailed in the Results, with Geisser-Greenhouse correction for sphericity. Post hoc analyses were performed using Sidak’s multiple comparisons tests primarily, with Fisher’s LSD used for direct male/female comparisons on RR20/RI60 schedules; post hoc results are primarily reported on figures. Correlation analyses were evaluated via the Pearson correlation coefficient (*r*). Scatter plots were fit with a line (simple linear regression) or curve (nonlinear, model reported alongside results). Figures show means ± SEM.

## 3. Results

### 3.1. Self-administration training on RR20 or RI60

Rats were trained on a seeking-taking chained schedule of self-administration, with the seeking lever reinforced on either an RR20 or RI60 schedule during the final training stage. The ratios and intervals used for the RR20 and RI60 schedules, respectively, are unpredictable and randomly selected via a probability function (i.e., 0.05 probability per lever press for RR20 and 0.0166 probability per second for RI60). To illustrate the randomness of the schedules, we plotted the relative frequency (% of trials) for different ratios and intervals, using representative data from >1000 trials each for RR20 and RI60, and fit nonlinear curves to the data (Fig. S1a, b). In this data set, the largest ratio observed was 178 and the longest interval was 490 s. As expected, for the RR20 schedule ~5 % of trials were rewarded after one press and for the RI60 schedule ~1.6 % of trials were rewarded with a press after one second. We also plotted the cumulative frequency for different ratios and intervals and fit nonlinear curves (Fig. S1c,d). This data shows that for the RR20 schedule 62 % of trials had a ratio of 20 or below and for the RI60 schedule 64 % of trials had an interval of 60 or below.

**Fig. 1.**
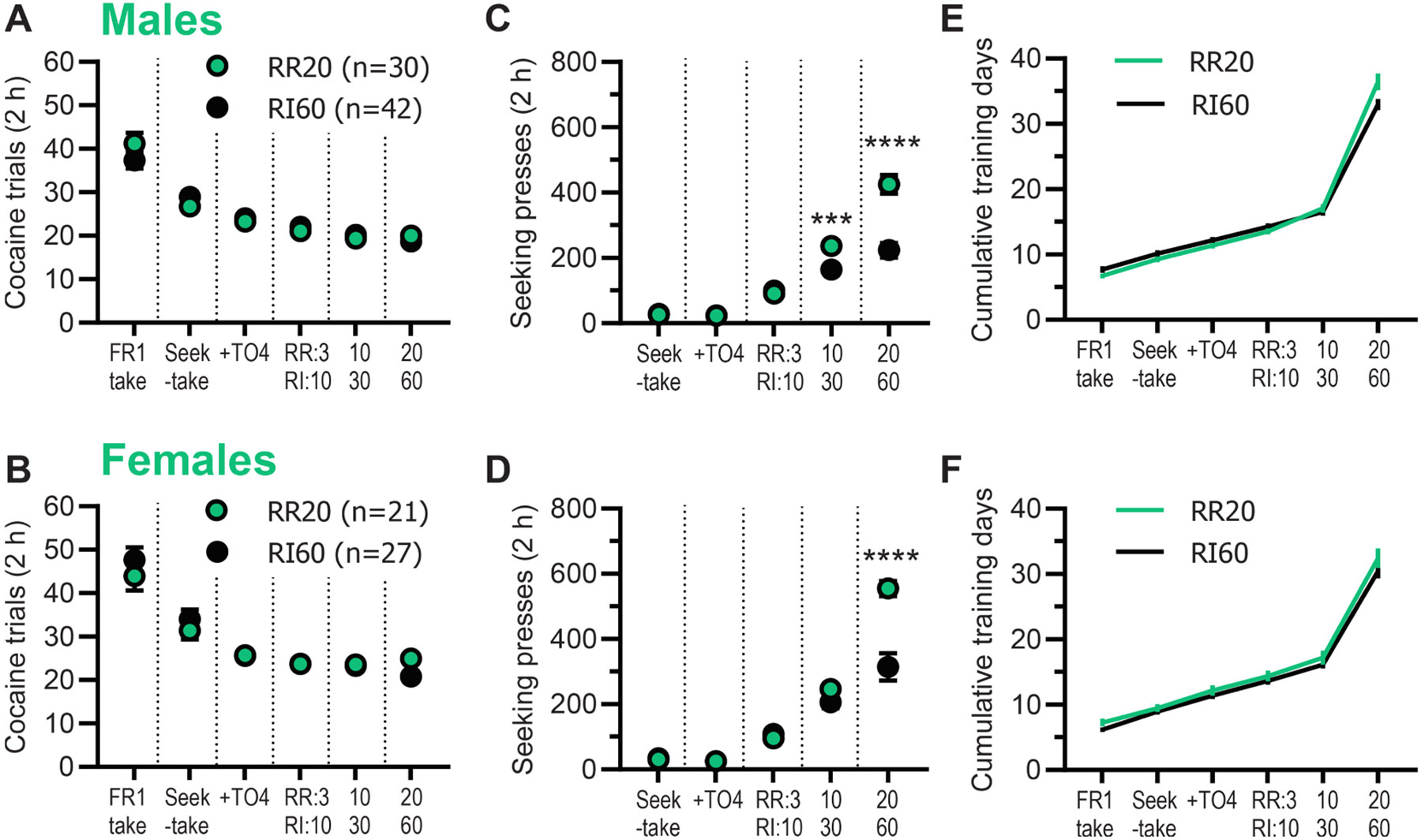
Cocaine self-administration training on RR20 and RI60 schedules. Data shown for different stages of training, including FR1 taking, FR1 seeking-taking, adding a 4-min timeout between trials (+TO4), RR3 or RI10 seeking-taking, RR10 or RI30 seeking-taking, and RR20 or RI60 seeking-taking. **(A–B)** Trials for each stage of training in males (A) and females (B) assigned to RR20 or RI60 schedules. **(C–D)** Seeking lever presses for each stage of training in males (C) and females (D). RR20 and RI60 schedules are different in the last stages of training (***p < 0.001, ****p < 0.0001). **(E–F)** Cumulative number of days in each training stage for males (E) and females (F).

For male and female rats trained to self-administer cocaine (2 h per day) on either an RR20 or RI60 schedule, we analyzed trial and seeking data across training by averaging per rat the last two sessions at each training stage and the last four sessions for the final training stage (RR20 or RI60). We found that the two schedules did not differ in the number of trials completed across training stages for males or females (Fig. 1a, b; 2-way ANOVA for Males: Schedule F_1,70_ = 0.0049, p = 0.94; Training F_1.89,132_ = 126, p < 0.0001; Schedule × Training interaction F_5,350_ = 2.73, p = 0.019; no significant post hoc effects between schedules; Females: Schedule F_1,46_ = 0.0429, p = 0.84; Training F_1.63,74.8_ = 81.7, p < 0.0001; Schedule × Training F_5,230_ = 1.91, p = 0.093). However, schedules were significantly different in terms of seeking presses in the final stages of training, with more seeking presses for RR20 in males and females (Fig. 1c, d; 2-way ANOVA for Males: Schedule F_1,70_ = 31.1, p < 0.0001; Training F_1.43,100_ = 228, p < 0.0001; Schedule × Training interaction F_4,280_ = 28.0, p < 0.0001; Females: Schedule F_1,46_ = 17.6, p = 0.0001; Training F_1.41,65.0_ = 187, p < 0.0001; Schedule × Training interaction F_4,184_ = 18.0, p < 0.0001; post hoc effects shown on figures). There was no difference between schedules in terms of the number of sessions prior to punishment, with males averaging 34 days and females averaging 31 days, although a significant interaction in males reflects more training days at the final stage for the RR20 schedule (Fig. 1e, f; 2-way ANOVA for Males: Schedule F_1,70_ = 0.0175, p = 0.90; Training F_1.28,89.6_ = 1086, p < 0.0001; Schedule × Training interaction F_5,350_ = 8.40, p < 0.0001; Females: Schedule F_1,46_ = 1.16, p = 0.29; Training F_1.53,70.1_ = 1010, p < 0.0001; Schedule × Training interaction F_5,230_ = 0.901, p = 0.48). Direct comparison of males and females revealed that the total number of sessions prior to punishment was higher in males, particularly with the RR20 schedule (2-way ANOVA: Sex F_1,116_ = 5.64, p = 0.019; Schedule F_1,116_ = 8.12, p = 0.0052; Sex × Schedule interaction F_1,116_ = 0.387, p = 0.53).

**Fig. 2.**
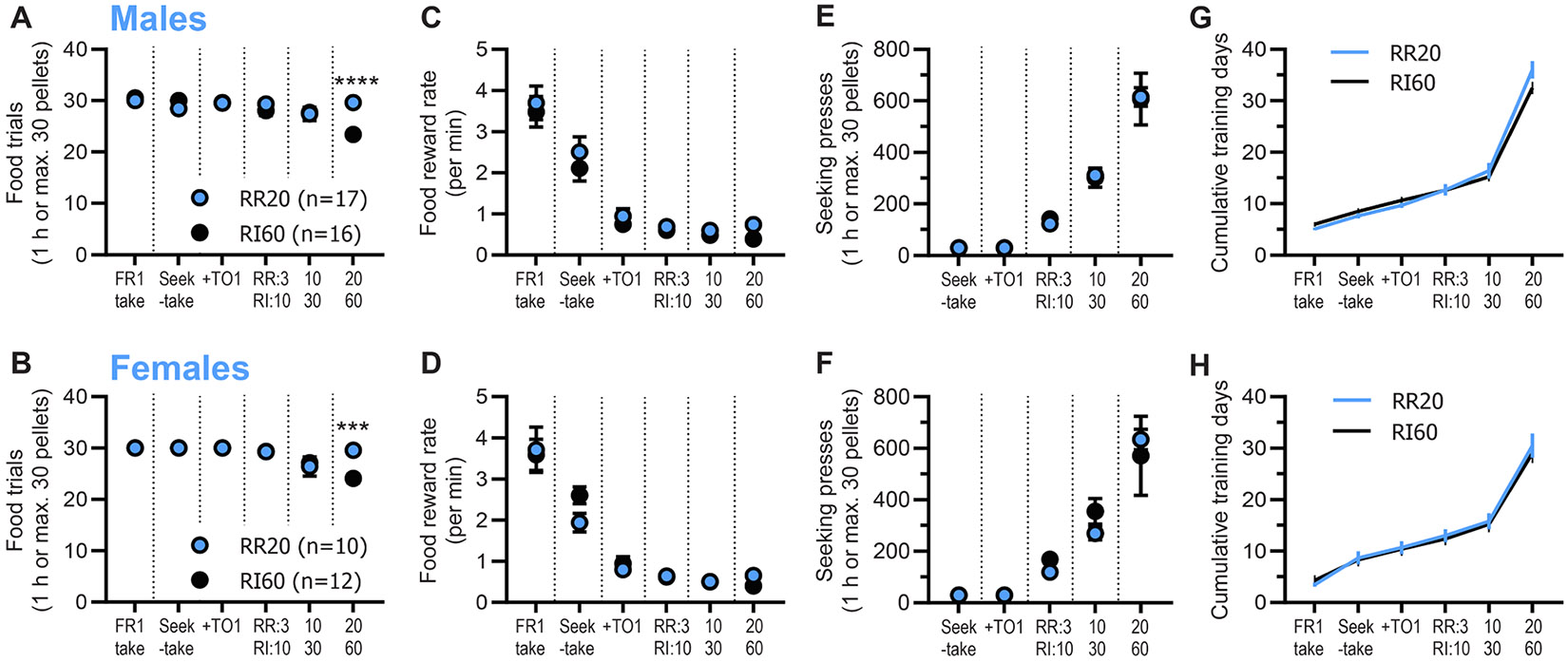
Food self-administration training on RR20 and RI60 schedules. Data shown for different stages of training, including FR1 taking, FR1 seeking-taking, adding a 1-min timeout between trials (+TO1), RR3 or RI10 seeking-taking, RR10 or RI30 seeking-taking, and RR20 or RI60 seeking-taking. **(A–B)** Trials for each stage of training in males (A) and females (B) assigned to RR20 or RI60 schedules. Sessions were capped at 30 trials or 1 h. RR20 and RI60 schedules are different in the last stage of training (***p < 0.001, ****p < 0.0001). **(C–D)** Reward rate (trials per min) for each stage of training in males (C) and females (D). **(E–F)** Seeking lever presses for each stage of training in males (E) and females (F). **(G–H)** Cumulative number of days in each training stage for males (G) and females (H).

Separate groups of male and female rats were trained to self-administer food pellets (1 h per day or maximum 30 pellets), with the seeking lever reinforced on either an RR20 or RI60 schedule. The two schedules differed in the number of trials completed at the final training stage, with more trials for RR20 for males and females (Fig. 2a, b; 2-way ANOVA for Males: Schedule F_1,31_ = 2.74, p = 0.11; Training F_3.22,99.9_ = 7.61, p < 0.0001; Schedule × Training interaction F_5,155_ = 7.74, p < 0.0001; Females: Schedule F_1,20_ = 2.55, p = 0.13; Training F_1.61,32.2_ = 9.30, p = 0.0013; Schedule × Training interaction F_5,100_ = 5.09, p = 0.0003). Because food sessions ended after 1 h or after 30 pellets, whichever occurred first, we also assessed reward rate (pellets per min) to compare the schedules. Schedules did not differ in reward rate for either males or females (Fig. 2c, d; 2-way ANOVA for Males: Schedule F_1,31_ = 2.02, p = 0.17; Training F_2.08,64.6_ = 72.7, p < 0.0001; Schedule × Training interaction F_5,155_ = 0.175, p = 0.97; Females: Schedule F_1,20_ = 0.376, p = 0.55; Training F_1.52,30.4_ = 68.9, p < 0.0001; Schedule × Training interaction F_5,100_ = 1.07, p = 0.38). Schedules also did not differ in terms of seeking presses across training for either males or females (Fig. 2e, f; 2-way ANOVA for Males: Schedule F_1,31_ = 0.0011, p = 0.97; Training F_1.34,41.4_ = 96.8, p < 0.0001; Schedule × Training interaction F_4,124_ = 0.0514, p = 1.0; Females: Schedule F_1,20_ = 0.110, p = 0.74; Training F_1.19,23.8_ = 38.2, p < 0.0001; Schedule × Training interaction F_4,80_ = 0.524, p = 0.72). Finally, there was no difference between schedules in terms of the number of sessions prior to punishment, with males averaging 34 days and females averaging at 30 days, although a significant interaction in males reflects more training days at the final stage for the RR20 schedule (Fig. 2g, h; 2-way ANOVA for Males: Schedule F_1,31_ = 0.118, p = 0.73; Training F_2.02,62.6_ = 461, p < 0.0001; Schedule × Training interaction F_5,155_ = 3.28, p = 0.0077; no significant post hoc effects between schedules; Females: Schedule F_1,20_ = 0.0515, p = 0.82; Training F_1.83,36.6_ = 296, p < 0.0001; Schedule × Training interaction F_5,100_ = 0.526, p = 0.76). Direct comparison of males and females revealed that the total number of sessions prior to punishment was higher in males (2-way ANOVA: Sex F_1,51_ = 6.57, p = 0.013; Schedule F_1,51_ = 2.03, p = 0.16; Sex × Schedule interaction F_1,51_ = 0.325, p = 0.57).

### 3.2. Punishment of cocaine self-administration

**Fig. 3.**
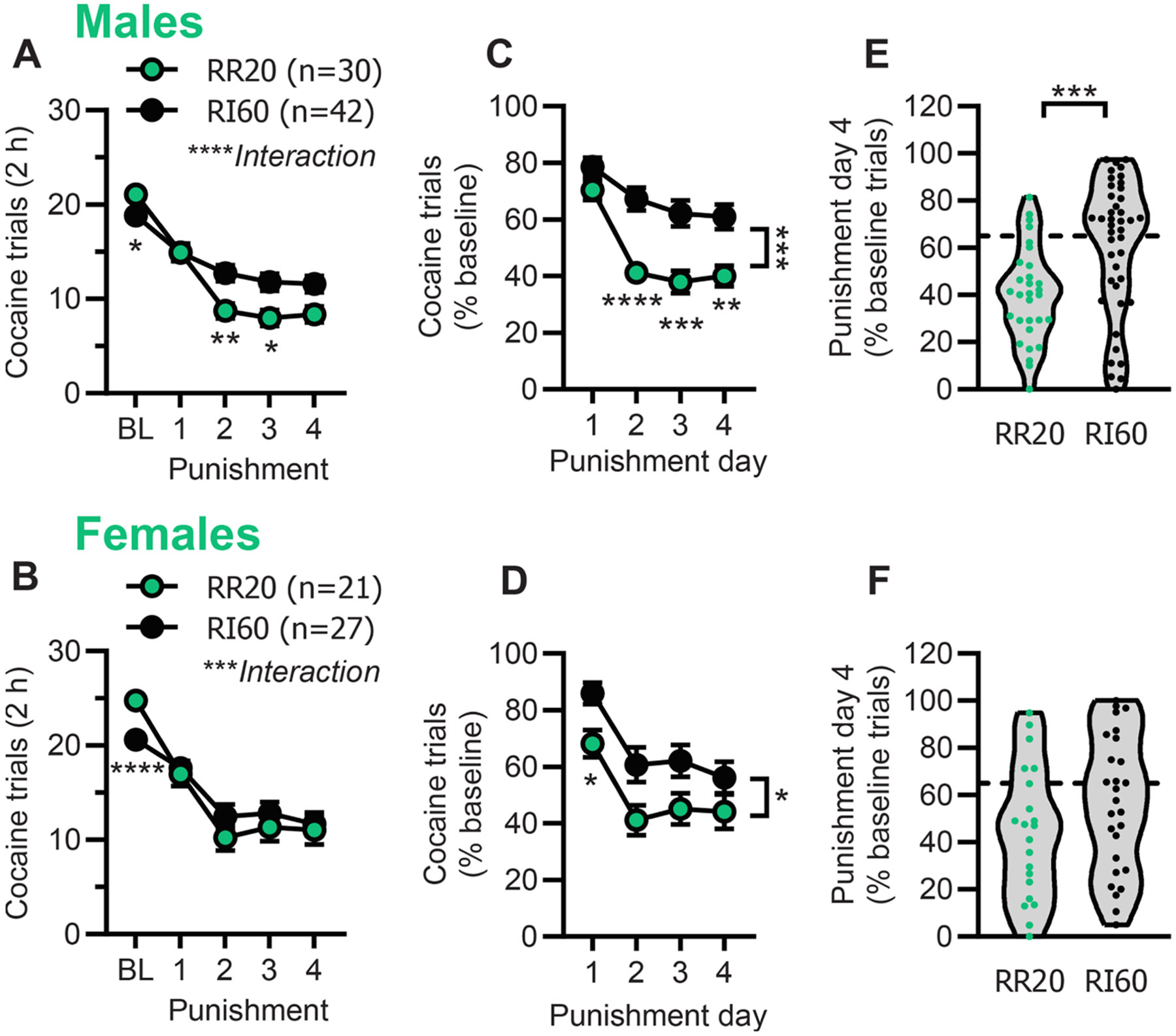
Punishment effects on cocaine trials. **(A–B)** Cocaine trials completed on RR20 or RI60 schedule during seeking-taking baseline (BL; average of four sessions prior to punishment) and the four days of punishment in males (A) and females (B). Significant Schedule × Day interactions are noted, as well as significant post hoc differences between schedules. **(C–D)** Trials on the four days of punishment as a percent of baseline for males (C) and females (D). Main effects and post hoc differences between schedules are shown. **(E–F)** Violin plots show trials on the fourth day of punishment as a percent of baseline. *p < 0.05, **p < 0.01, ***p < 0.001, ****p < 0.0001

After ≥13 days (avg. 16 days) on the final seeking-taking schedule for cocaine, rats were exposed to four days of punishment testing. For each rat, an average of the four days before punishment was used as a baseline to assess the effects of punishment. We observed a significant reduction in trials over the four days of punishment, as well as a significant interaction with schedule that indicated greater suppression with RR20, in males and females (Fig. 3a, b; 2-way ANOVA for Males: Day F_2.76,194_ = 149, p < 0.0001; Schedule F_1,70_ = 3.31, p = 0.073; Day × Schedule interaction F_4,280_ = 15.0, p < 0.0001; Females: Day F_2.82,130_ = 90.6, p < 0.0001; Schedule F_1,46_ = 0.0158, p = 0.90; Day × Schedule interaction F_4,184_ = 5.70, p = 0.0002; all post hoc effects shown on figures). Male rats trained on RI60 completed less trials during baseline but completed more trials during punishment (which means more cocaine infusions and more footshocks), indicating greater punishment resistance (Fig. 3a). Female rats trained on RI60 completed less trials during baseline but completed similar trials during punishment, also indicating greater punishment resistance with RI60 (Fig. 3b). As a percent of baseline, male rats trained on RR20 had a greater reduction in trials across the four days of punishment, as compared to RI60 (Fig. 3c; 2-way ANOVA: Schedule F_1,70_ = 15.9, p = 0.0002; Punishment Day F_2.39,167_ = 51.0, p < 0.0001; Schedule × Punishment Day interaction F_3,210_ = 6.13, p = 0.0005). Female rats trained on RR20 also had a greater reduction in trials across the four days of punishment (Fig. 3d; Schedule F_1,46_ = 5.85, p = 0.020; Punishment Day F_2.61,120_ = 37.2, p < 0.0001; Schedule × Punishment Day interaction F_3,138_ = 0.573, p = 0.63). Violin plots for the fourth day of punishment revealed greater punishment resistance with RI60 in males, but no difference in females (Fig. 3e, f; *t*-test for Males: t_70_ = 3.46, p = 0.0009; Females: t_46_ = 1.46, p = 0.15). A dashed line is plotted at 65 % to indicate the threshold for punishment resistance in individual rats, based on our previous work with a larger population of male rats exposed to punishment and k-means clustering analysis (Jones et al., 2024). Together, these results indicate increased punishment resistance on the RI60 schedule when comparing cocaine trials for males and females. Direct comparison of males and females revealed a difference in baseline trials (2-way ANOVA: Sex F_1,116_ = 25.5, p < 0.0001; Schedule F_1,116_ = 34.4, p < 0.0001; Sex × Schedule interaction F_1,116_ = 2.37, p = 0.13), with females completing more trials on RR20 (p < 0.0001) and RI60 (p = 0.0085). There was no sex difference when comparing fourth day of punishment as a percent of baseline, although a main effect of schedule indicates greater resistance with RI60 (2-way ANOVA: Sex F_1,116_ = 0.0044, p = 0.95; Schedule F_1,116_ = 10.9, p = 0.0013; Sex × Schedule interaction F_1,116_ = 0.768, p = 0.38). Analysis of all four days of punishment (as a percent of baseline) revealed a 3-way interaction for Sex × Schedule × Day (Fig. 3c, d; F_3,348_ = 2.67, p = 0.048), likely due to a Schedule × Day interaction in males but not females (as revealed by 2-way analyses above).

**Fig. 4.**
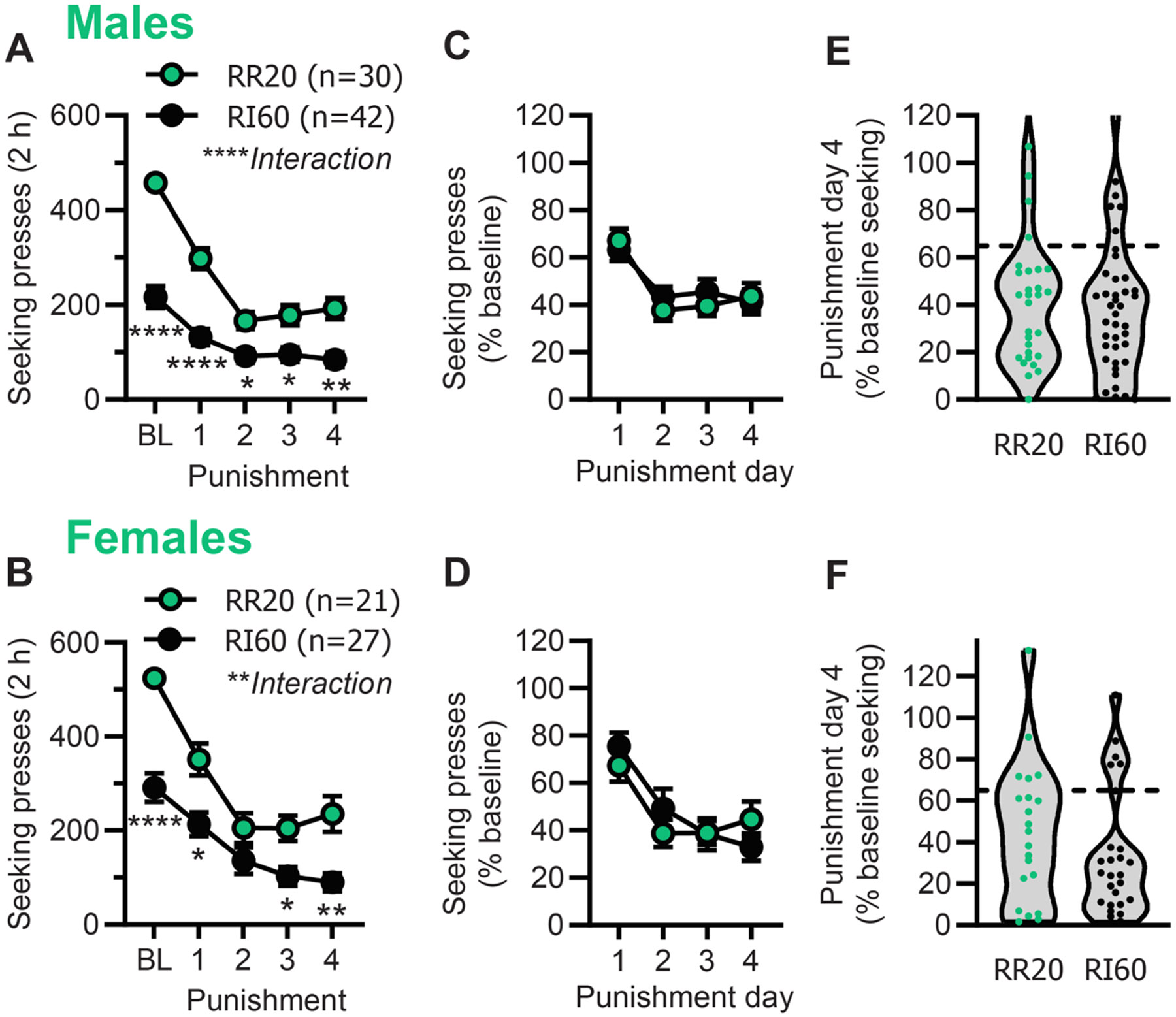
Punishment effects on cocaine seeking. **(A–B)** Cocaine seeking presses on RR20 or RI60 schedule during seeking-taking baseline (BL; average of four sessions prior to punishment) and the four days of punishment in males (A) and females (B). Significant Schedule × Day interactions are noted, as well as significant post hoc differences between schedules. **(C–D)** Seeking presses on the four days of punishment as a percent of baseline for males (C) and females (D). **(E–F)** Violin plots show seeking on the fourth day of punishment as a percent of baseline. *p < 0.05, **p < 0.01, ****p < 0.0001.

We also evaluated the effects of punishment on the number of seeking responses, as opposed to the number of trials completed. We observed a significant reduction in seeking presses over the four days of punishment, as well as a significant interaction with schedule that indicated greater suppression with RR20 as compared to RI60 in both males and females (Fig. 4a, b; 2-way ANOVA for Males: Day F_2.51,176_ = 82.1, p < 0.0001; Schedule F_1,70_ = 42.6, p < 0.0001; Day × Schedule interaction F_4,280_ = 12.6, p < 0.0001; Females: Day F_3.04,140_ = 57.5, p < 0.0001; Schedule F_1,46_ = 22.5, p < 0.0001; Day × Schedule interaction F_4,184_ = 4.52, p = 0.0016). RI60 training led to fewer seeking presses as compared to RR20 at baseline and during punishment in males and females (Fig. 4a, b). As a percent of baseline, we saw a similar reduction in seeking across the four days of punishment for RR20 and RI60 in males and females, although a significant interaction in females reflects that RR20-trained rats reached maximum sensitivity by Day 2 whereas RI60-trained rats were most sensitive on Day 4 (Fig. 4c, d; 2-way ANOVA for Males: Schedule F_1,70_ = 0.0495, p = 0.82; Punishment Day F_2.65,186_ = 25.7, p < 0.0001; Schedule × Punishment Day interaction F_3,210_ = 1.23, p = 0.30; Females: Schedule F_1,46_ = 0.0417, p = 0.84; Punishment Day F_2.67,123_ = 28.0, p < 0.0001; Schedule × Punishment Day interaction F_3,138_ = 2.92, p = 0.037). Violin plots for the fourth day of punishment showed similar punishment sensitivity with RR20 and RI60 in both males and females (Fig. 4e, f; *t*-test for Males: t_70_ = 0.308, p = 0.76; Females: t_46_ = 1.28, p = 0.21). Direct comparison of males and females revealed a difference in seeking presses at baseline (2-way ANOVA: Sex F_1,116_ = 8.21, p = 0.0049; Schedule F_1,116_ = 93.7, p < 0.0001; Sex × Schedule interaction F_1,116_ = 0.0321, p = 0.85), with females making more presses than males on RI60 (p < 0.05). There was no sex difference in seeking on the fourth day of punishment as a percent of baseline (2-way ANOVA: Sex F_1,116_ = 0.351, p = 0.55; Schedule F_1,116_ = 1.37, p = 0.25; Sex × Schedule interaction F_1,116_ = 0.598, p = 0.44). Analysis of all four days of punishment (as a percent of baseline) revealed no 3-way interaction for Sex × Schedule × Day (Fig. 4c, d; F_3,348_ = 1.79, p = 0.15). Altogether, these data show that cocaine self-administration under the RI60 schedule led to greater resistance to punishment in males and females in terms of cocaine trials but not seeking presses.

### 3.3. Punishment of food self-administration

**Fig. 5.**
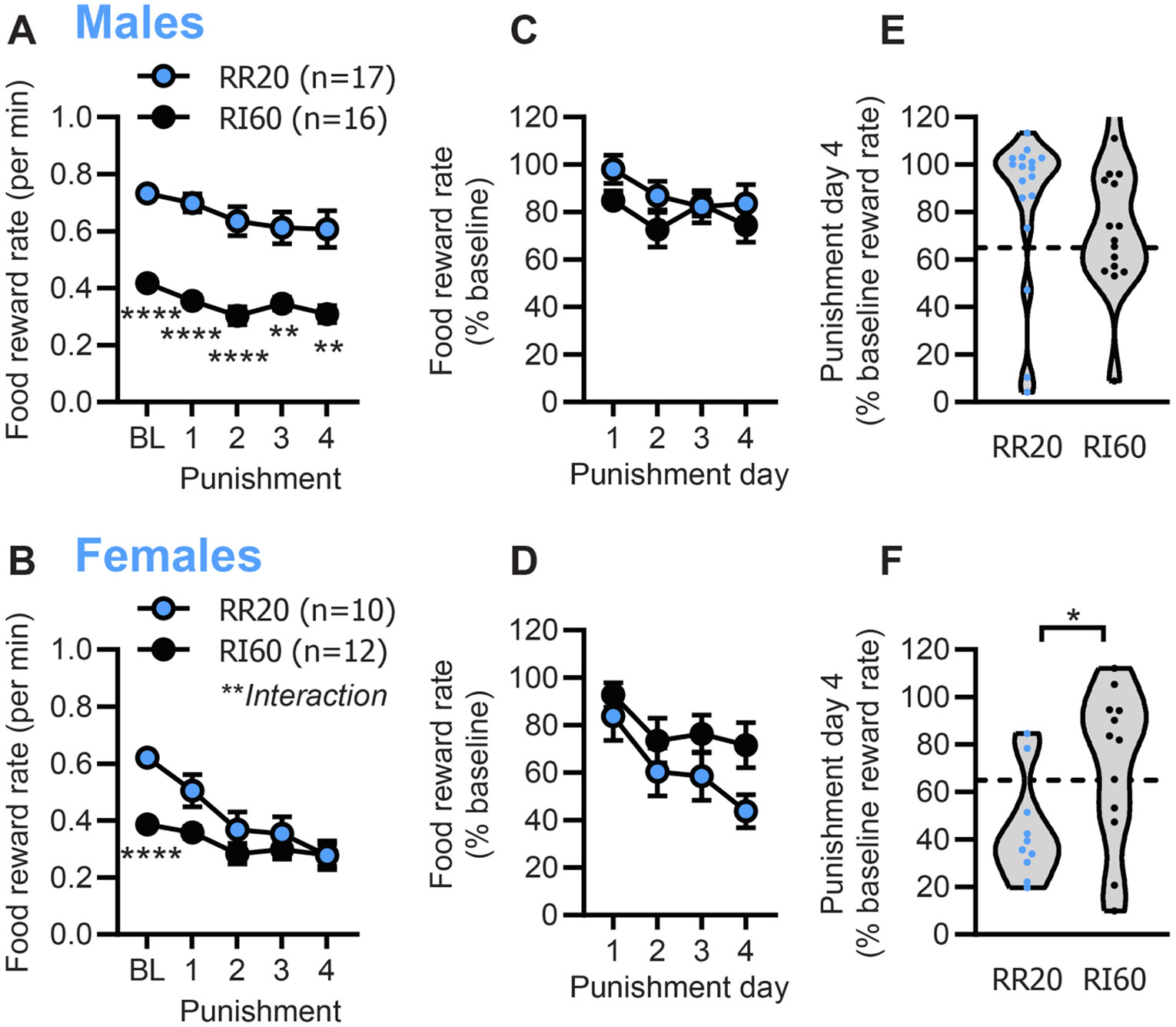
Punishment effects on food reward rate. **(A–B)** Food reward rate (trials per min) on RR20 or RI60 schedule during seeking-taking baseline (BL; average of four sessions prior to punishment) and the four days of punishment in males (A) and females (B). Significant Schedule × Day interactions are noted, as well as significant post hoc differences between schedules. **(C–D)** Reward rate on the four days of punishment as a percent of baseline for males (C) and females (D). **(E–F)** Violin plots show reward rate on the fourth day of punishment as a percent of baseline. *p < 0.05, **p < 0.01, ****p < 0.0001.

After ≥12 days (avg. 17 days) on the final seeking-taking schedule for food, rats were exposed to four days of punishment testing. We observed a significantly higher food reward rate with the RR20 schedule and found a significant reduction in food reward rate over the four days of punishment in males and females; a significant interaction in females reflects greater suppression with RR20 (Fig. 5a, b; 2-way ANOVA for Males: Day F_2.92,90.6_ = 6.92, p = 0.0003; Schedule F_1,31_ = 50.4, p < 0.0001; Day × Schedule interaction F_4,124_ = 0.671, p = 0.61; Females: Day F_3.13,62.5_ = 18.9, p < 0.0001; Schedule F_1,20_ = 5.58, p = 0.029; Day × Schedule interaction F_4,80_ = 4.54, p = 0.0024; post hoc effects shown on figures). As a percent of baseline, we saw similar reward rates across the four days of punishment for RR20 and RI60 in both males and females (Fig. 5c, d; 2-way ANOVA for Males: Schedule F_1,31_ = 1.73, p = 0.20; Punishment Day F_2.67,82.7_ = 2.74, p = 0.055; Schedule × Punishment Day interaction F_3,93_ = 1.02, p = 0.39; Females: Schedule F_1,20_ = 2.54, p = 0.13; Punishment Day F_2.72,54.3_ = 12.1, p < 0.0001; Schedule × Punishment Day interaction F_3,60_ = 1.18, p = 0.32). Violin plots for the fourth day of punishment showed that most male rats appeared resistant to punishment for food regardless of schedule, whereas female rats were more resistant with RI60 as compared to RR20 (Fig. 5e, f; *t*-test for Males: t_31_ = 0.873, p = 0.39; Females: t_20_ = 2.28, p = 0.033). Direct comparison of males and females revealed a difference in baseline reward rates (2-way ANOVA: Sex F_1,51_ = 9.35, p = 0.0035; Schedule F_1,51_ = 140, p < 0.0001; Sex × Schedule interaction F_1,51_ = 3.04, p = 0.088), with males showing a faster reward rate on RR20 (p = 0.0016). There was also a sex difference when comparing fourth day of punishment as a percent of baseline (2-way ANOVA: Sex F_1,51_ = 6.76, p = 0.012; Schedule F_1,51_ = 1.26, p = 0.27; Sex × Schedule interaction F_1,51_ = 5.10, p = 0.028), with males showing more punishment resistance on RR20 (p = 0.0015). Analysis of all four days of punishment (as a percent of baseline) revealed no 3-way interaction for Sex × Schedule × Day (Fig. 5c, d; F_3,153_ = 0.690, p = 0.56).

**Fig. 6.**
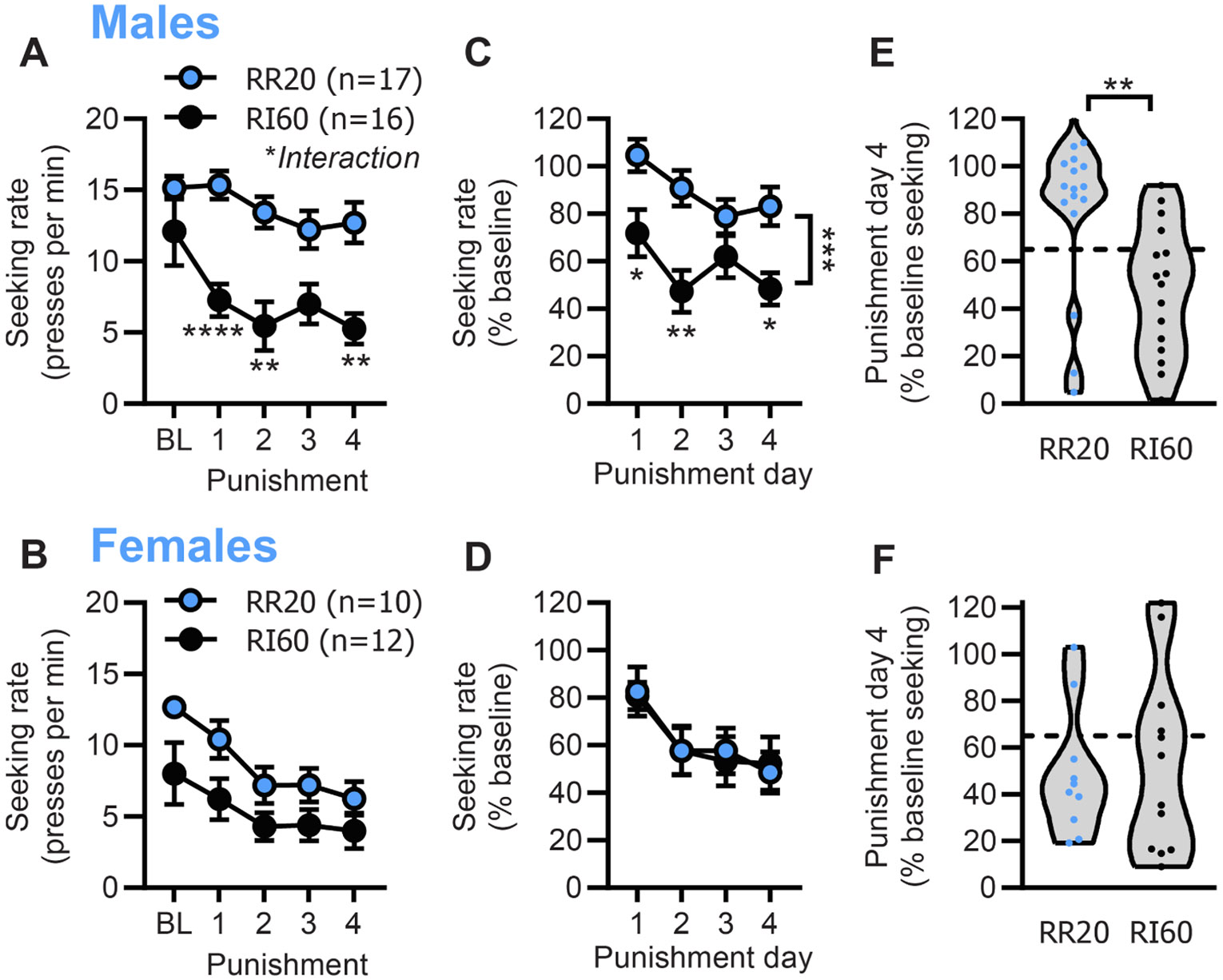
Punishment effects on food seeking. **(A–B)** Food seeking rate (presses per min) on RR20 or RI60 schedule during seeking-taking baseline (BL; average of four sessions prior to punishment) and the four days of punishment in males (A) and females (B). Significant Schedule × Day interactions are noted, as well as significant post hoc differences between schedules. **(C–D)** Seeking rate on the four days of punishment as a percent of baseline for males (C) and females (D). Main effects and post hoc differences between schedules are shown. **(E–F)** Violin plots show seeking rate on the fourth day of punishment as a percent of baseline. *p < 0.05, **p < 0.01, ***p < 0.001, ****p < 0.0001. [1.5-column width].

We also evaluated the effects of punishment on seeking responses for food. We saw a significantly higher seeking rate with the RR20 schedule and saw a significant reduction in seeking rate over the four days of punishment in both males and females; a significant interaction in males reflects greater suppression with the RI60 schedule (Fig. 6a, b; 2-way ANOVA for Males: Day F_2.93,90.8_ = 9.31, p < 0.0001; Schedule F_1,31_ = 15.5, p = 0.0004; Day × Schedule interaction F_4,124_ = 3.03, p = 0.020; Females: Day F_2.19,43.8_ = 11.0, p < 0.0001; Schedule F_1,20_ = 5.21, p = 0.034; Day × Schedule interaction F_4,80_ = 0.581, p = 0.68). As a percent of baseline, the RI60 schedule caused greater reductions in seeking rate over the four days of punishment in males as compared to RR20, whereas RR20 and RI60 led to similar reductions in seeking rate in females (Fig. 6c, d; 2-way ANOVA for Males: Schedule F_1,31_ = 13.3, p = 0.0010; Punishment Day F_2.26,69.9_ = 5.88, p = 0.0031; Schedule × Punishment Day interaction F_3,93_ = 1.77, p = 0.16; Females: Schedule F_1,20_ = 0.0038, p = 0.95; Punishment Day F_2.22,44.3_ = 9.22, p = 0.0003; Schedule × Punishment Day interaction F_3,60_ = 0.137, p = 0.94). Violin plots for the fourth day of punishment revealed greater punishment resistance in terms of seeking with RR20 in males as compared to RI60 but similar sensitivity for RR20 and RI60 in females (Fig. 6e, f; *t*-test for Males: t_31_ = 3.26, p = 0.0027; Females: t_20_ = 0.256, p = 0.80). Direct comparisons of males and females revealed no difference in seeking rate at baseline (2-way ANOVA: Sex F_1,51_ = 3.30, p = 0.075; Schedule F_1,51_ = 4.56, p = 0.038; Sex × Schedule interaction F_1,51_ = 0.197, p = 0.66), but a difference in seeking rate on the fourth day of punishment as a percent of baseline (2-way ANOVA: Sex F_1,51_ = 3.02, p = 0.088; Schedule F_1,51_ = 3.09, p = 0.085; Sex × Schedule interaction F_1,51_ = 4.76, p = 0.034), with greater punishment resistance in males on RR20 as compared to females (p = 0.0090). Analysis of all four days of punishment (as a percent of baseline) revealed no 3-way interaction for Sex × Schedule × Day (Fig. 6c, d; F_3,153_ = 1.13, p = 0.34).

We found that male rats gained more weight than female rats over the course of the food study. At the start of self-administration training, males weighed 370 g on average and females weighed 280 g. By the first day of punishment testing, males weighed 470 g (100 g weight gain) and females weighed 300 g (20 g weight gain).

Therefore, like cocaine, food self-administration under the RI60 schedule led to greater resistance to punishment in females in terms of reward rate but not seeking rate. However, unlike cocaine, males self-administering food showed resistance to punishment on both the RI60 and RR20 schedules in terms of reward rate, and the RI60 schedule led to increased sensitivity to punishment in terms of seeking rate.

### 3.4. Response rate vs. reward rate

**Fig. 7.**
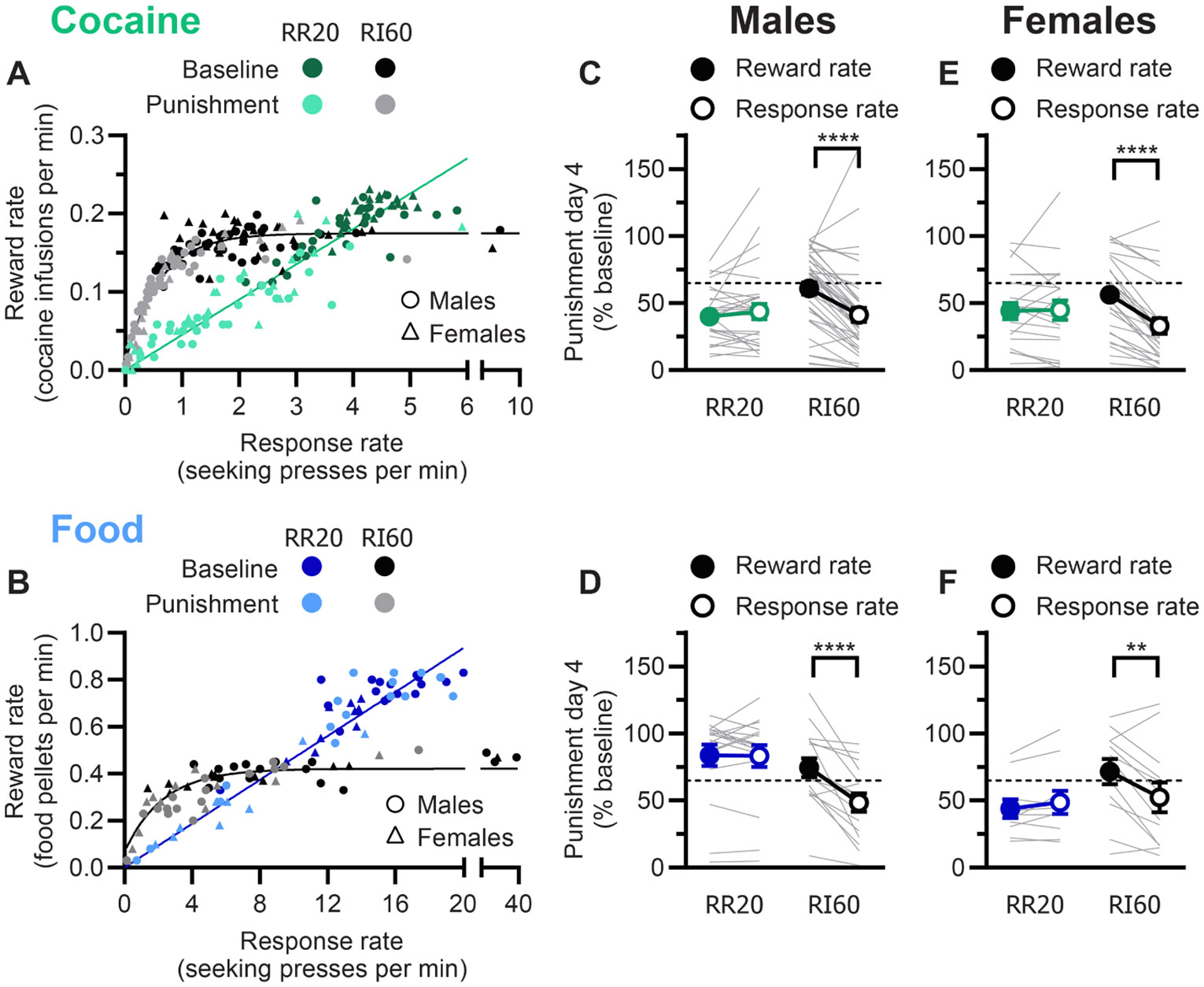
Relation between response rate and reward rate for RR20 and RI60 schedules. **(A–B)** Scatter plots of response rate and reward rate for cocaine (A) and food (B) for baseline self-administration (average of 4 days prior to punishment) and the fourth day of punishment in males (circles) and females (triangles) on RR20 or RI60 schedules. Best-fit lines for RR20 and curves for RI60 were created using larger data sets with all four baseline sessions and four punishment sessions. **(C–F)** Comparisons of reward rate and response rate on the fourth day of punishment as a percent of baseline for cocaine (C) and food (D) in males, and for cocaine (E) and food (F) in females. Response rate was suppressed to a greater degree than reward rate on the RI60 schedule. **p < 0.01, ****p < 0.0001.

For both cocaine and food self-administration, we found that punishment resulted in dissociated effects on trials (i.e., reward rate) and seeking (i.e., response rate) for the RI60 schedule but not the RR20 schedule. In other words, punishment caused a greater suppression of seeking presses than reward rate on the RI60 schedule. This dissociation between trials and seeking can be understood by examining scatter plots of response rate vs. reward rate for baseline self-administration (average of 4 days prior to punishment) and the last punishment session (fourth day) in males and females and comparing the RR20 and RI60 schedules for cocaine (Fig. 7a) and food (Fig. 7b). To better understand the relation between response rate and reward rate, we determined the best-fit models for each schedule using larger data sets that included all four sessions prior to punishment and all four sessions of punishment. Data from RR20-trained rats self-administering cocaine revealed a linear regression, indicating that as response rate decreased, reward rate decreased in a similar manner (linear regression: F_1,373_ = 7150, p < 0.0001; correlation: r = 0.89, p < 0.0001; Fig. 7a). In contrast, data from RI60-trained rats self-administering cocaine generated a nonlinear regression, indicating that as response rate decreased, reward rate decreased but to a lesser extent (exponential plateau model: r = 0.91; comparison of fit against a line: F_1,517_ = 1238, p < 0.0001; Fig. 7a). Similarly, data from male and female rats self-administering food revealed a linear regression for the RR20 schedule (linear regression: F_1,223_ = 6182, p < 0.0001; correlation: r = 0.85, p < 0.0001; Fig. 7b) and a nonlinear regression for the RI60 schedule (exponential plateau model: r = 0.75; comparison of fit against a line: F_1,205_ = 135, p < 0.0001; Fig. 7b).

Therefore, on the RI60 schedule we observed greater reductions in response rate than reward rate on the final session of punishment, while on the RR20 schedule we observed similar reductions in response rate and reward rate. We observed this in male rats self-administering cocaine or food (Fig. 7c, d; 2-way ANOVA for Cocaine: Schedule F_1,70_ = 2.01, p = 0.16; Rate F_1,70_ = 10.7, p = 0.0017; Schedule × Rate interaction F_1,70_ = 22.5, p < 0.0001; Food: Schedule F_1,31_ = 4.67, p = 0.039; Rate F_1,31_ = 18.3, p = 0.0002; Schedule × Rate interaction F_1,31_ = 16.8, p = 0.0003). We also observed this in female rats self-administering cocaine or food (Fig. 7e, f; Cocaine: Schedule F_1,46_ = 0.00042, p = 0.98; Rate F_1,46_ = 22.1, p < 0.0001; Schedule × Rate interaction F_1,46_ = 24.8, p < 0.0001; Food: Schedule F_1,20_ = 1.49, p = 0.24; Rate F_1,20_ = 3.75, p = 0.067; Schedule × Rate interaction F_1,20_ = 10.3, p = 0.0045). This dissociation between response rate and reward rate can be explained by examining the curve generated by the RI60 schedule and simplifying it into two linear functions that intersect at an inflection point. Reductions in response rate to the right of the inflection point (when reward rate plateaus) result in minor reductions in reward rate. However, reductions in response rate to the left of the inflection point result in drastic reductions in reward rate, more steeply than the RR20 schedule even. Therefore, RI60-trained rats might experience two different relations between response rate and reward rate, depending on their baseline response rate and its proximity to the inflection point.

### 3.5. Punishment influence by other factors

**Fig. 8.**
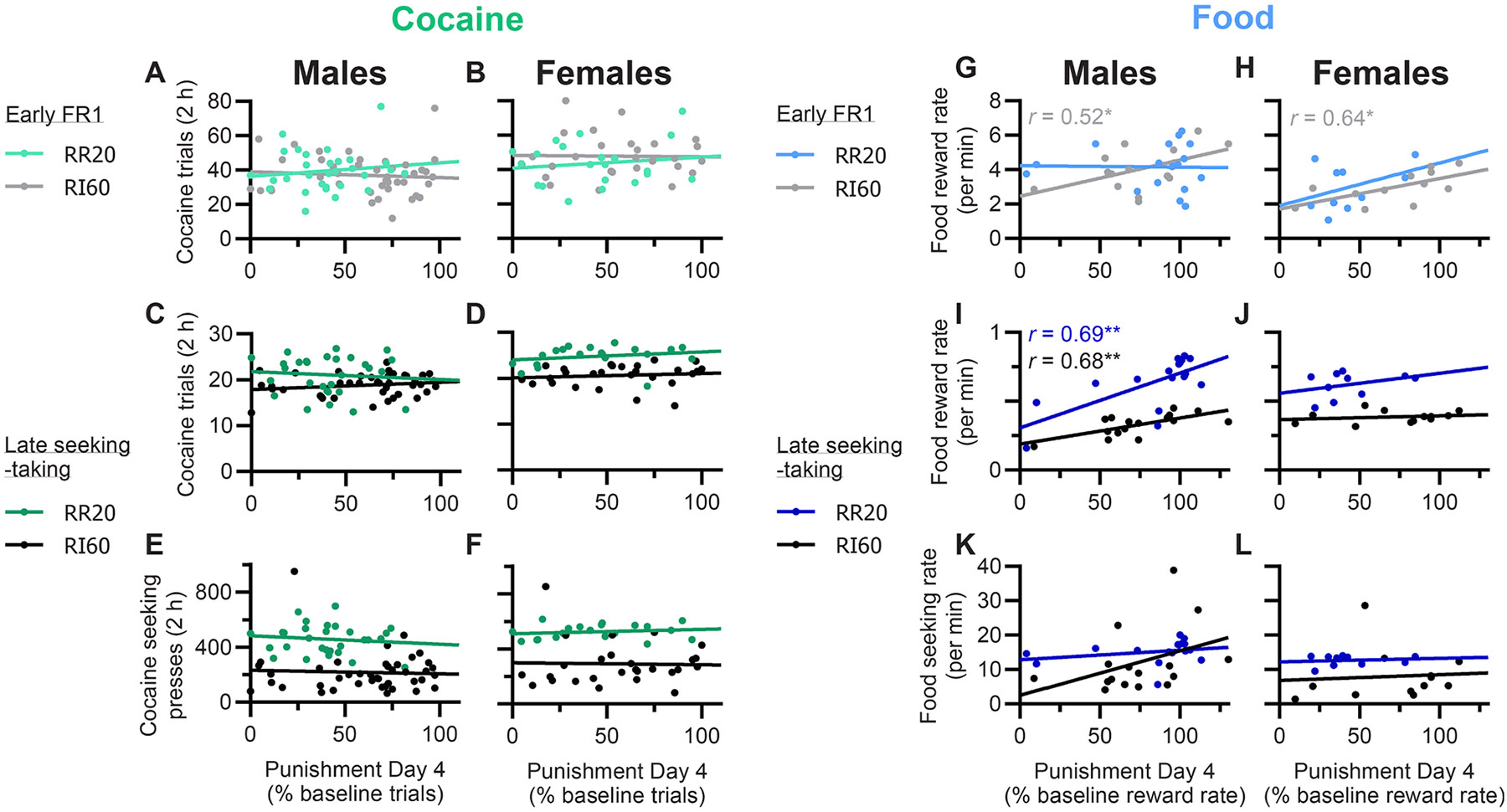
Relation between punishment resistance and baseline self-administration for RR20 and RI60 schedules. For all scatter plots, the x-axis shows the fourth day of punishment as a percentage of baseline trials (for cocaine) or reward rate (for food) and the y-axis shows baseline self-administration measures. **(A–B)** Cocaine trials during early FR1 self-administration in males (A) and females (B). **(C–D)** Cocaine trials during seeking-taking self-administration in males (C) and females (D). **(E–F)** Cocaine seeking presses during seeking-taking self-administration in males (E) and females (F). **(G–H)** Food reward rate (trials per min) during early FR1 self-administration in males (G) and females (H), which is correlated with punishment resistance for the RI60 schedule. **(I–J)** Food reward rate during seeking-taking self-administration in males (I) and females (J), which is correlated with punishment resistance in males for RR20 and RI60 schedules. **(K–L)** Food seeking rate (presses per min) during seeking-taking self-administration in males (K) and females (L). *p < 0.05, **p < 0.01.

We evaluated whether punishment resistance (Day 4 trials as percent of baseline) was correlated with baseline self-administration measures, including reward rate during early FR1 training, reward rate during the final stage of seeking-taking, or response rate during seeking-taking. For cocaine, in both RR20 and RI60 schedules, we found no correlations in males or females between punishment resistance and trials during FR1 self-administration (average of last two FR1 sessions; Fig. 8a, b; Males RR20: r = 0.13, p = 0.49; Males RI60: r = −0.084, p = 0.60; Females RR20: r = 0.14, p = 0.54; Females RI60: r= −0.020, p = 0.92) or trials during seeking-taking (average of last four sessions prior to punishment; Fig. 8c, d; Males RR20: r = −0.083, p = 0.66; Males RI60: r = 0.19, p = 0.23; Females RR20: r = 0.21, p = 0.35; Females RI60: r = 0.11, p = 0.57). Additionally, we found no correlations with seeking presses during seeking-taking (average of last four sessions prior to punishment; Fig. 8e, f; Males RR20: r = −0.12, p = 0.54; Males RI60: r = −0.049, p = 0.76; Females RR20: r = 0.17, p = 0.46; Females RI60: r = −0.026, p = 0.90). This indicates that punishment resistance on the RI60 schedule cannot be explained by baseline differences in seeking, and thus, cannot be explained by the dissociation between response rate and reward rate.

For food, we found correlations between punishment resistance on the RI60 schedule and reward rate during FR1 self-administration in males (RI60: r = 0.52, p = 0.041; RR20: r = −0.020, p = 0.94; Fig. 8g) and females (RI60: r = 0.64, p = 0.026; RR20: r = 0.41, p = 0.24; Fig. 8h), indicating that punishment resistance on RI60 might be related to baseline food motivation. We also found correlations with reward rate during seeking-taking self-administration for both RR20 and RI60 schedules in males (RR20: r = 0.69, p = 0.0023; RI60: r = 0.68, p = 0.0037; Fig. 8i) but not females (Fig. 8j; RR20: r = 0.34, p = 0.33; RI60: r = 0.21, p = 0.52), indicating that punishment resistance for food in males may be related to enhanced food motivation. We found no significant correlations with seeking rate during seeking-taking in males or females (Fig. 8k, l; Males RR20: r = 0.27, p = 0.29; Males RI60: r = 0.38, p = 0.14; Females RR20: r = 0.15, p = 0.68; Females RI60: r = 0.074, p = 0.82), again indicating that punishment resistance on the RI60 schedule cannot be ascribed to baseline differences in seeking rate.

We also investigated a possible influence of estrous cycle on punishment resistance for cocaine or food. For cocaine-trained females, we analyzed the four days of punishment (trials as percent of baseline) according to estrous cycle stage and found a main effect of cycle stage for the RR20 schedule, with rats in proestrus appearing more sensitive across days (Fig. S2a; Mixed-effects model: Cycle F_3,56_ = 3.51, p = 0.021; Punishment Day F_2.00,37.3_ = 6.68, p = 0.0033; Cycle × Punishment Day F_9,56_ = 0.717, p = 0.69; although no significant post hoc effects across cycle stages). We found no effect of cycle stage for the RI60 schedule (Fig. S2b; Mixed-effects model: Cycle F_3,31_ = 0.421, p = 0.74; Punishment Day F_2.39,42.2_ = 4.77, p = 0.0097; Cycle × Punishment Day F_9,53_ = 0.546, p = 0.83). In food-trained females, we observed no effects of cycle stage for the RR20 or RI60 schedule (Fig. S2c, d; Mixed-effects model for RR20: Cycle F_2,9_ = 0.0841, p = 0.92; Punishment Day F_1.36,5.42_ = 3.90, p = 0.096; Mixed-effects model for RI60: Cycle F_1,11_ = 0.336, p = 0.57; Punishment Day F_3,11_ = 1.51, p = 0.26).

### 3.6. Cocaine vs. Food reward

Direct comparison of cocaine vs. food self-administration showed more seeking presses per trial for the RI60 schedule for food as compared to cocaine in both males and females (Fig. S3a, b; 2-way ANOVA for Males: Reward F_1,112_ = 44.3, p < 0.0001; Punishment F_1,112_ = 23.0, p < 0.0001; Reward × Punishment interaction F_1,112_ = 4.63, p = 0.034; Females: Reward F_1,74_ = 7.88, p = 0.0064; Punishment F_1,74_ = 13.5, p = 0.0004; Reward × Punishment interaction F_1,74_ = 0.0021, p = 0.96). We did not compare seeking presses per trial for the RR20 schedule because the schedule dictates an average of 20 presses per trial. When comparing punishment effects on reward rate for cocaine vs. food (Day 4 as a percent of baseline), we found that males were more resistant for food than cocaine, particularly with the RR20 schedule, whereas females showed similar resistance for food and cocaine (Fig. S3c, d; 2-way ANOVA for Males: Reward F_1,101_ = 25.2, p < 0.0001; Schedule F_1,101_ = 1.03, p = 0.31; Reward × Schedule interaction F_1,101_ = 7.00, p = 0.0095; Females: Reward F_1,66_ = 1.06, p = 0.31; Schedule F_1,66_ = 7.34, p = 0.0086; Reward × Schedule interaction F_1,66_ = 1.13, p = 0.29). When comparing punishment effects on seeking rate, we again found that males were more resistant for food than cocaine, particularly with the RR20 schedule, whereas females showed similar resistance for food and cocaine (Fig. S3e, f; Males: Reward F_1,101_ = 12.0, p = 0.0008; Schedule F_1,101_ = 7.58, p = 0.0070; Reward × Schedule interaction F_1,101_ = 5.75, p = 0.018; Females: Reward F_1,66_ = 1.91, p = 0.17; Schedule F_1,66_ = 0.230, p = 0.63; Reward × Schedule interaction F_1,66_ = 0.855, p = 0.36).

## 4. Discussion

We observed greater punishment resistance with the RI60 schedule as compared to the RR20 schedule. For cocaine self-administration, the RI60 schedule led to more trials completed during punishment as compared to baseline trials in males and females. For food self-administration, the RI60 schedule led to higher reward rates during punishment as compared to baseline reward rates in female rats. Male rats appeared punishment resistant for food on both RI60 and RR20 schedules. We found a dissociation between punishment effects on reward rate and response rate with the RI60 schedule, such that seeking responses were suppressed to a greater degree than trials. However, we determined that the classification of punishment resistance was more aptly based on trials (i.e., reward rate), rather than seeking responses, because rewards and footshocks were delivered according to trials.

### 4.1. RR and RI schedule differences

With cocaine self-administration, we observed increased punishment resistance with the RI60 schedule, as compared to the RR20 schedule, when comparing trials completed. The RI60 schedule yielded some rats that were punishment resistant and others that were punishment sensitive, supporting the common use of RI schedules for studying punishment of cocaine seeking (Chen et al., 2013; Jonkman et al., 2012a, 2012b; Limpens et al., 2014, 2015; Pelloux et al., 2007, 2012, 2015; Vanderschuren & Everitt, 2004; Zhou et al., 2019). In contrast, the RR20 schedule yielded mostly punishment-sensitive rats. Although previous studies have not investigated RR schedules with cocaine punishment, an FR5 schedule of reinforcement has been used to drive punishment resistance in a subset of rats (Belin et al., 2011, 2008, 2009; Cannella et al., 2013; Deroche-Gamonet et al., 2004; Kasanetz et al., 2010, 2013). RR20 and FR5 are both ratio schedules but they differ in several ways including predictability and effort. The RR20 schedule is known to bias toward goal-directed responding, which might increase sensitivity to punishment, while it is unknown whether FR5-trained rats would be goal-directed or habitual. Previous work with FR5 also included extended cocaine self-administration prior to punishment testing and this alone may drive increased punishment resistance regardless of schedule. We found increased punishment resistance with RI60 when compared to RR20 in a between-subjects design. It is unknown whether rats trained on both RR and RI schedules in different contexts would also show within-subject schedule-related differences in punishment resistance, similar to how previous work showed within-subject schedule-related differences in goal-directed and habitual responding (Gremel & Costa, 2013). Further, it is unknown whether schedule-related differences would persist at different shock intensities, probabilities, or delays. Future studies addressing these questions may provide insight into the influence of schedules on punishment resistance.

With food self-administration, we again found that the RI60 schedule led to greater punishment resistance but only in female rats. The majority of male rats were punishment resistant in terms of reward rate as compared to baseline on both RR20 or RI60 schedules. This may indicate that punishment resistance for food in male rats is associated with goal-directed food seeking, and our previous work supports this (Jones et al., 2024). Likewise, Seiler et al. (2022) saw greater punishment resistance (i.e., more shocks received) with the RI60 schedule in mice responding for sucrose via nosepokes. Seiler et al. also showed a higher baseline reward rate with RI60 as compared to RR20, which may indicate that punishment resistance is related to greater food motivation, as reflected by higher reward rates. In support of this, we found positive correlations between baseline food reward rates and punishment resistance for both RR20 and RI60 schedules in male rats (Fig. 8i). However, we found no significant correlations for reward rate and punishment resistance in food-trained females or cocaine-trained males or females. Further, we observed lower baseline reward rates with RI60 in rats responding for cocaine or food, even though RI60 was associated with greater punishment resistance overall. Altogether, these results indicate that baseline differences in reward rate cannot fully account for punishment differences between schedules.

Although we found greater punishment resistance for cocaine with the RI60 schedule in terms of trials, we observed a similar footshock-induced suppression of seeking responses when comparing the RR20 and RI60 schedules. Thus, rats trained on RI60 showed significantly greater reductions in their response rates as compared to their reward rates during punishment. We observed this effect across sexes and reward types. Although Chen et al. (2013) reported no suppression of seeking responses for cocaine in punishment-resistant rats, Pelloux et al. (2007) saw greater suppression of cocaine seeking than trials in punishment-resistant rats trained on RI. Seiler et al. (2022) observed a similar result in mice self-administering sucrose, such that the RI60 schedule led to higher reward rates (and footshock delivery) than the RR20 schedule during punishment despite a similar suppression of responding with RI60 and RR20 schedules. Thus, results from previous studies parallel our finding that footshock punishment caused greater suppression of response rate than reward rate with the RI60 schedule. This dissociation between response rate and reward rate with the RI60 schedule can be explained by the mechanics of the schedules (i.e., the underlying mechanisms of reinforcement delivery). However, as discussed below, the dissociation between response rate and reward rate on the RI60 schedule does not explain the enhanced occurrence of punishment resistance on this schedule.

RR and RI schedules differ in terms of the association between response and reward. RR schedules require an unpredictable number of responses, whereas interval schedules require a response after an unpredictable amount of time has elapsed. Thus, reward delivery on RR schedules is directly related to rate of responding, creating a linear relation between response rate and reward rate (Baum, 1973; Dickinson, 1985; Perez & Dickinson, 2020). In contrast, reward delivery on RI schedules requires only one response after a specified amount of time. With an RI schedule, increases in response rate lead to increases in reward rate only to an extent before reaching an asymptote (Baum, 1973), creating a nonlinear relation between response rate and reward rate (Fig. 7). The same is true when considering the relation between response rate and footshock rate for RR and RI schedules. On the RI schedule, the nonlinear relation means that a reduction in seeking responses can occur without a reduction in trials (or with a minimal reduction in trials), particularly when the baseline seeking rate is higher. However, punishment resistance on the RI60 schedule cannot be explained by schedule mechanics alone because baseline seeking rate was not correlated with punishment resistance (Fig. 8). If we had observed a positive correlation between these measures for RI60, then this might indicate that punishment resistance on RI60 is an artifact of the schedule, with high responders showing reductions in seeking without concomitant reductions in trials, and low responders consistently showing reductions in seeking and trials. Instead, we found that high and low responders could both be sensitive or resistant to punishment, indicating that punishment resistance on the RI60 schedule is not an artifact of schedule mechanics. Thus, a dissociation between response rate and reward rate on the RI60 schedule is observed for both reinforcement and punishment, and it is explained by the mechanics of the schedule, but it does not directly explain or fully account for the enhanced occurrence of punishment resistance on the RI60 schedule.

### 4.2. Punishment resistance and habits

Enhanced punishment resistance with the RI60 schedule may indicate a role for habits. Compulsive drug use has long been theorized to stem from a loss of control over habitual behavior, making habits maladaptive and inflexible (Belin et al., 2013; Brown et al., 2022; Everitt & Robbins, 2005, 2016; Everitt, 2014; Ostlund & Balleine, 2008; Smith & Laiks, 2018). Punishment resistance for alcohol and cocaine has been shown to involve the dorsolateral striatum, an area critical for habitual behavior (Giuliano et al., 2019, 2021; Jonkman et al., 2012a). Recent studies showed that habits are typically flexible and can be overridden by goal-directed control under certain conditions (Bouton, 2021; Bouton, Broomer, Rey, & Thrailkill, 2020; Steinfeld & Bouton, 2020; Trask, Shipman, Green, & Bouton, 2020). Therefore, habits may only become maladaptive when they can no longer be overruled by goal-directed control. In support of this, we recently showed that although habitual responding for cocaine did not predict punishment resistance, punishment resistance was related to the continued use of habits under conditions that typically elicit increased goal-directed control (Jones et al., 2024). In that work, we assessed goal-directed and habitual responding using outcome devaluation via cocaine satiety (Jones et al., 2022, 2024). Although we did not detect a difference between RR20 and RI60 schedules in terms of punishment resistance in the previous study (Jones et al., 2024), our current work with larger group sizes revealed significant differences between RR20 and RI60 schedules. Taken together with our previous work, these findings indicate that punishment resistance for cocaine is greater on the RI60 schedule and is related to the continued use of habitual behavior.

RI schedules appear to bias habitual responding and punishment resistance, although the underlying mechanisms are uncertain. Various mechanisms have been hypothesized to explain why RR and RI schedules bias goal-directed and habitual behavior. For example, it has been hypothesized that the nonlinear relation between response rate and reward rate on RI schedules creates a weaker response-outcome (R-O) association, biasing for habits (Dickinson, 1985; Perez & Dickinson, 2020). RI schedules have also been hypothesized to bias habitual responding due to longer average intervals between responses and outcomes (i.e., poor R-O contiguity) (DeRusso et al., 2010; Garr, Bushra, Tu, & Delamater, 2020; Handel & Smith, 2024). These same mechanisms may also drive increased punishment resistance with RI schedules. For a stimulus to be effective as a punisher, strong R-O contiguity is known to be particularly critical because contingent shocks delivered with a delay have little effect on responding (Azrin, 1956; Camp, Raymond, & Church, 1967; Liley, Gabriel, Sable, & Simon, 2019). Longer RO intervals on the RI schedule may introduce uncertainty about the response-punisher association (Campbell & Church, 1969), which may also affect perceived R-O contingency. Recent work from Jean-Richard-Dit-Bressel et al. (2021), Jean-Richard-Dit-Bressel, Ma, Bradfield, Killcross, and McNally (2019), Jean-Richard-Dit-Bressel et al. (2023) has implicated impaired contingency detection in punishment insensitivity. Therefore, a reduced perception of R-O contingency might bias habitual responding and punishment resistance. It is possible that separate features of the RI schedule bias habits and punishment resistance. In support of this, Seiler et al. (2022) found that the RI60 schedule led to increased punishment resistance and decreased behavioral flexibility (as assessed via an omission test with negative contingency), but that the two measures were not correlated. In our previous work we also found no correlation between punishment resistance and pre-punishment habitual responding, although we found a positive correlation with post-punishment habits, demonstrating that habitual responding and punishment resistance share some common mechanisms but are not completely intertwined (Jones et al., 2024).

Although we generally found greater punishment resistance with the RI60 schedule, we also observed resistance with the RR20 schedule in some circumstances. In particular, male rats had a tendency to show punishment resistance for food, even when trained on the RR20 schedule. This may implicate goal-directed responding in some types of punishment resistance, as suggested by previous studies (Hogarth, 2020; Singer, Fadanelli, Kawa, & Robinson, 2018). Accordingly, our recent work found that most male rats with punishment resistance for food showed goal-directed responding and some rats with punishment resistance for cocaine were goal-directed as well (Jones et al., 2024). Further, Seiler et al. (2022) found punishment resistance for food was more strongly tied to dopamine activity in the dorsomedial striatum, an area necessary for goal-directed responding. Therefore, habitual behavior is not necessary for punishment resistance.

### 4.3. Reward and sex differences

We found sex differences in baseline self-administration of cocaine and food, even before the introduction of footshock punishment. Female rats showed a higher reward rate for cocaine self-administration at baseline with both RR20 and RI60 schedules, whereas male rats had a higher reward rate for food self-administration at baseline with the RR20 schedule. This is similar to what Seiler et al. (2022) observed for sucrose seeking in males, with higher baseline reward rates and punishment resistance in males as compared to females.

We found reward and sex differences in punishment resistance as well. Whereas female rats showed similar punishment resistance for food and cocaine, male rats were more resistant for food than cocaine, similar to previous findings (Jones et al., 2024). In particular, male rats self-administering food on the RR20 schedule were more resistant than females on the RR20 schedule, and more resistant than males self-administering cocaine on the RR20 schedule. Sex differences in punishment sensitivity for food might be related to increased food motivation in males, evidenced by greater weight gain than females across the course of the study and higher reward rate with the RR20 schedule. In addition, previous work showed that male rats work harder than females to earn food rewards, even despite footshock risk (Chowdhury et al., 2019; Jacobs & Moghaddam, 2020; Orsini, Willis, Gilbert, Bizon, & Setlow, 2016). We found positive correlations between baseline food reward rate and punishment resistance in males for both schedules, further indicating that punishment resistance for food-trained males may be associated with motivation. However, when considering punishment resistance for cocaine, we found no correlations with baseline self-administration or responding, indicating that resistance cannot be explained by differences in reinforcement. Additionally, we previously showed that punishment resistance could not be attributed to differences in footshock sensitivity (Jones et al., 2024). Similarly, recent work investigating individual variability in humans and animals found that punishment insensitivity could not be explained by increased reward sensitivity or decreased aversion sensitivity (Jean-Richard-dit-Bressel et al., 2023, 2019, 2021). Therefore, reward sensitivity/motivation may be a factor in some types of punishment resistance (e.g., for food in male rats), but does not appear to play a major role in other types of punishment resistance (e.g., for cocaine).

Finally, it is worth noting that our observations of punishment resistance with food are in contrast with previous studies that did not observe punishment resistance with food, even after extended sucrose or chow self-administration (Limpens et al., 2014; Pelloux et al., 2007, 2015; Radke et al., 2017; Vanderschuren & Everitt, 2004). In addition, previous studies observed punishment resistance for cocaine with extended but not limited exposure to cocaine self-administration (Deroche-Gamonet et al., 2004; Jonkman et al., 2012b; Pelloux et al., 2007, 2015; Xue, Steketee, & Sun, 2012), whereas the rats in the current studies received moderate exposure. These discrepancies between studies may be attributed to differences in methods, such as footshock intensity, reward size and type (45-mg pellet vs. liquid sucrose/saccharin), omission of reward on footshock trials, schedule of reinforcement, the criteria for resistance, and studying suppression vs. conditioned suppression. Future studies may investigate how extended self-administration affects the influence of reinforcement schedule on punishment resistance.

### 4.4. Conclusions

Overall, these results indicate that schedules of reinforcement influence sensitivity to punishment. Therefore, the schedule of reinforcement should be carefully considered when designing studies involving punishment. Further investigation comparing different schedules may provide insight into the mechanisms of punishment resistance, as well as compulsive responding.

## Supplementary Material

Supplementary figures

## Appendix A. Supplementary material

Supplementary data to this article can be found online at https://doi.org/10.1016/j.nlm.2024.107961.
