# Supplementary material for: Random interval schedule of reinforcement influences punishment resistance for cocaine in rats
Source: Neurobiol Learn Mem. Author manuscript; Available in PMC 2026 Feb 14. (PMC12904990; doi:10.1016/j.nlm.2024.107961)
Supplement: Supplementary figures [file NIHMS2143142-supplement-Supplementary_figures.pdf]

Supplementary Figures

Random interval schedule of reinforcement influences punishment resistance for cocaine in rats

Bradley O. Jones, Haley F. Spencer, Adelis M. Cruz, Morgan S. Paladino, Sophia N. Handel, Rachel J. Smith

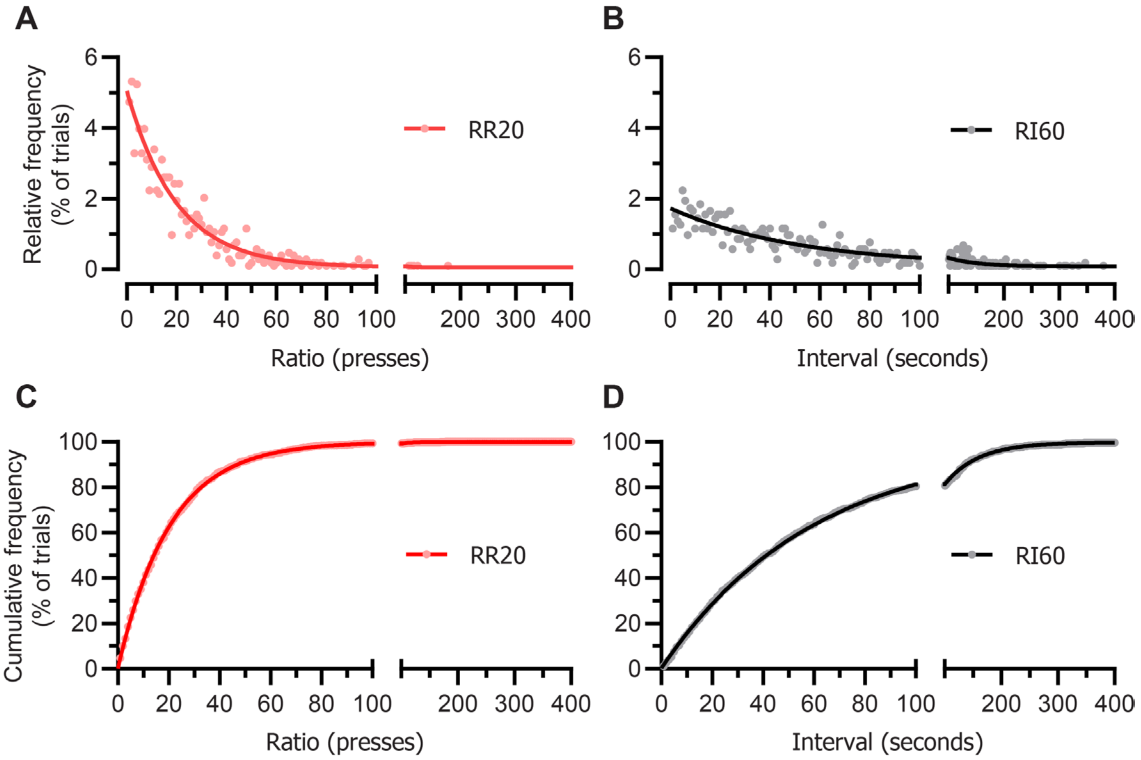

Fig. S1 | Frequency distributions of ratios and intervals randomly selected on the RR20 and RI60 schedules. A-B) Relative frequency distribution (percent of trials) for different ratios on the RR20 schedule (A) and different intervals on the RI60 schedule (B), based on a data set of >1000 trials for each schedule. The RR20 schedule was reinforced after a given number of presses (A), whereas the RI60 schedule was reinforced when a lever press occurred after a given interval of time (B). Values of zero were not plotted to make non-zero values more easily seen. Nonlinear curves were fit to the RR20 data (one phase decay model, goodness of fit: $r^2 = 0.93$) and RI60 data ($r^2 = 0.83$). **C-D)** Cumulative frequency distribution for ratios on RR20 (C) and intervals on RI60 (D). Nonlinear curves were fit to the RR20 data (goodness of fit: $r^2 = 0.99$) and RI60 data ($r^2 = 0.99$).

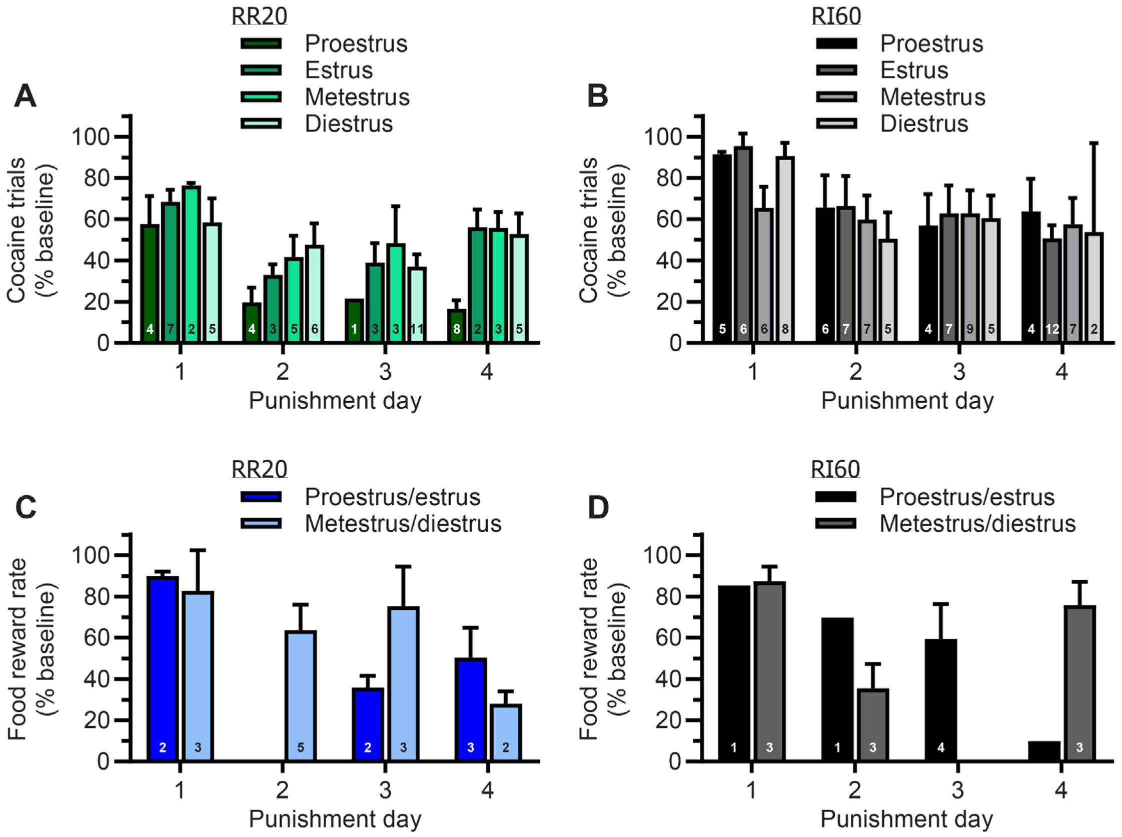

Fig. S2 | Estrous cycle interactions with punishment resistance. A-B) Four days of punishment (trials as percent of baseline) for females trained to self-administer cocaine on RR20 (A) or RI60 (B) schedules. Rats were categorized according to the estrous cycle stage on each day (proestrus, estrus, metestrus, or diestrus). **C-D)** Four days of punishment (reward rate as percent of baseline) for females trained to self-administer food on RR20 (C) or RI60 (D) schedules. Rats were categorized according to the estrous cycle stage on each day (due to lower numbers, rats were grouped together for proestrus/estrus or metestrus/diestrus).

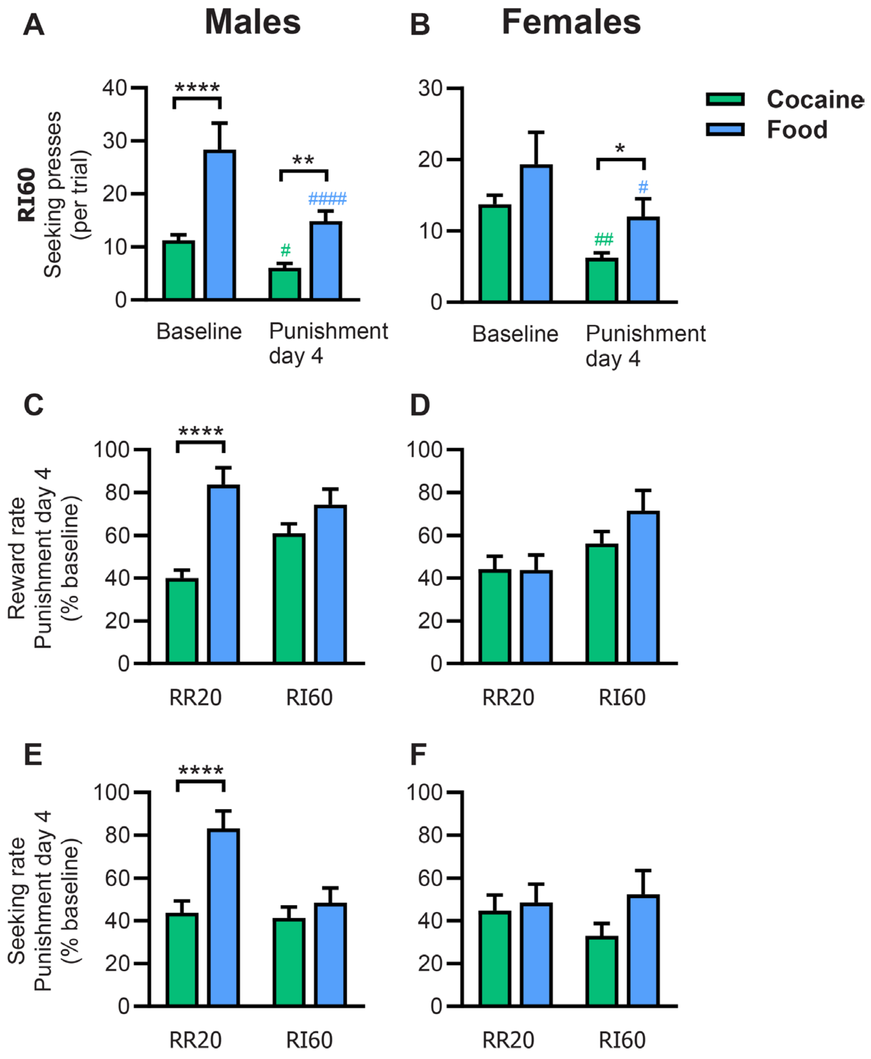

Fig. S3 | Comparisons of cocaine and food self-administration at baseline and during punishment. A-B) Comparisons of cocaine and food in terms of seeking presses per trial for the RI60 schedule in males (A) and females (B). Food-trained rats showed more seeking presses per trial as compared to cocaine-trained rats (post hoc differences: $*p < 0.05$, $**p < 0.01$, $****p < 0.0001$). All groups showed a significant reduction on the fourth day of punishment as compared to baseline seeking-taking (post hoc differences: $^{\#}p < 0.05$, $^{\#\#}p < 0.01$, $^{\#\#\#\#}p < 0.0001$). **C-F)** Comparisons of cocaine and food on the fourth day of punishment (as a percent of baseline) in terms of reward rate in males (C) and females (D), as well as seeking rate in males (E) and females (F). Male rats showed greater punishment resistance for food than cocaine, particularly on the RR20 schedule ($****p < 0.0001$).
